# Implementing a digital comprehensive myopia prevention and control strategy for children and adolescents in China: a cost-effectiveness analysis

**DOI:** 10.1016/j.lanwpc.2023.100837

**Published:** 2023-07-13

**Authors:** Ruyue Li, Kaiwen Zhang, Shi-Ming Li, Yue Zhang, Jiaxin Tian, Zhecheng Lu, Huiqi Li, Liyuan Wang, Xiuhua Wan, Fengju Zhang, Li Li, Zi-Bing Jin, Ningli Wang, Hanruo Liu

**Affiliations:** a Beijing Institute of Ophthalmology, Beijing, 100730, China; b Beijing Tongren Eye Center, Beijing Tongren Hospital, Capital Medical University, Beijing, 100730, China; c School of Medical Technology, Beijing Institute of Technology, Beijing, 102488, China; d Department of Ophthalmology, The First Affiliated Hospital of Harbin Medical University, Harbin, 150000, China; e Department of Ophthalmology, National Key Discipline of Pediatrics, Ministry of Education, Beijing Children's Hospital, Capital Medical University, Beijing, 100000, China; f National Institute of Health Data Science at Peking University, Beijing, 100000, China

**Keywords:** Myopia, Digital, Cost-effectiveness, Health education, Screening

## Abstract

**Background:**

Children and adolescents’ myopia is a major public problem. Although the clinical effect of various interventions has been extensively studied, there is a lack of national-level and integral assessments to simultaneously quantify the economics and effectiveness of comprehensive myopia prevention and control programs. We aimed to compare the cost-effectiveness between traditional myopia prevention and control strategy, digital comprehensive myopia prevention and control strategy and school-based myopia screening program in China.

**Methods:**

A Markov model was used to compare the cost-utility and cost-effectiveness among school-based myopia screening, traditional myopia prevention and control strategy, and digital comprehensive myopia prevention and control strategy among 6 to 18-year-old rural and urban schoolchildren. Parameters were collected from published sources. The primary outcomes were quality-adjusted life-year, disability-adjusted life-year, incremental cost-utility ratio, and incremental cost-effectiveness ratio. Extensive sensitivity analyses were performed to test the robustness and sensitivity of base-case analysis.

**Findings:**

Compared with school-based myopia screening strategy, after implementing digital comprehensive myopia prevention and control strategy, the prevalence of myopia among 18-year-old students in rural and urban areas was reduced by 3.79% and 3.48%, respectively. The incremental cost-utility ratio per quality-adjusted life-year gained with the digital myopia management plan ($11,301 for rural setting, and $10,707 for urban setting) was less than 3 times the per capita gross domestic product in rural settings ($30,501) and less than 1 time the per capita gross domestic product in urban settings ($13,856). In cost-effectiveness analysis, the incremental cost-effectiveness ratio produced by digital comprehensive myopia management strategy ($37,446 and $41,814 per disability-adjusted life-year averted in rural and urban settings) slightly exceeded the cost-effectiveness threshold. When assuming perfect compliance, full coverage of outdoor activities and spectacles satisfied the cost-effectiveness threshold, and full coverage of outdoor activities produced the lowest cost ($321 for rural settings and $808 for urban settings).

**Interpretations:**

Health economic evidence confirmed the cost-effectiveness of promoting digital comprehensive myopia prevention and control strategies for schoolchildren at the national level. Sufficient evidence provides an economic and public health reference for further action by governments, policy-makers and other myopia-endemic countries.

**Funding:**

National Natural Science Foundation of China, NSFC (82171051), Beijing Natural Science Foundation (JQ20029), Capital Health Research and Development of Special (2020-2-1081), National Natural Science Foundation of China, NSFC (82071000), National Natural Science Foundation of China, NSFC (8197030562).

**Research in context**

**Evidence before this study**

We searched PubMed, MDLINE, CNKI and references listed in the identified papers for studies on the cost-effectiveness of myopia screening, prevention and treatment up to May 9, 2023. Previous studies have demonstrated the clinical efficiency and cost-effectiveness of single myopic intervention, focusing on vision test, low-concentration atropine, refractive surgery, and the treatment of pathologic myopia. However, the effect of a single intervention method is limited, and a comprehensive myopia prevention and control program can maximize the effects of each part (myopia screening, treatment and management). Nevertheless, economic evidence for comprehensive myopia prevention and control strategies targeting the full cycle of myopia progression in children and adolescents is lacking. Since the Chinese government has advocated a multi-party comprehensive myopia prevention and control strategy in recent years, including primary school-based screening, vision-related health education, follow-up treatment and digital management, it is necessary to assess the cost-effectiveness of a nationwide and digital comprehensive myopia prevention and control strategy in a myopia-endemic country such as China.

**Added value of this study**

To the best of our knowledge, this is the first cost-effectiveness analysis to assess the cost-utility and cost-effectiveness of implementing a digital comprehensive myopia prevention and control strategy. Our results showed that implementing a digital comprehensive myopia prevention and control strategy for 6–18-year-old children and adolescents is the most cost-effective option compared with traditional school-based myopia control programs in China. Among them, promoting full coverage of outdoor activities and spectacle wearing are the optimal option.

**Implications of all the available evidence**

The results of our study proved that the digital comprehensive myopia prevention and control strategy is the key to delaying the progression of myopia and preventing the occurrence of high myopia. It could save a lot of manpower and material resources, and realize the accessibility, affordability and personalized management of myopia prevention and control. The implementation of the project requires the joint efforts of the whole society, the government, schools, medical institutions, families and students. Among this progress, myopia-related health education can improve the knowledge of both students and parents and maximize the effects of other parts. Outdoor activity and wearing spectacles are the most cost-effective option to reduce the incidence and progression of myopia. The results of our study also provide an economic reference for the widespread promotion of highly cost-effective myopia management strategies in other countries.

## Introduction

Myopia is a major public health problem, the global prevalence of myopia was 30% in 2020, and it will rise to 50% by 2050.^1^^,^^2^ With rapid socioeconomic development, changes in lifestyle, and increased educational pressure, China is facing a situation of a high prevalence of myopia with an early onset age, high degree and rapid progress, especially among children and adolescents, who have an overall prevalence of 53.6%.^3^^,^^4^ High myopia leads to irreversible blindness, each additional 1D of myopia increases, the risk of myopic macular degeneration and retinal detachment increases by 58% and 30% respectively.^5^ A large number of unmet and under-met refractive error needs affect individual educational outcomes, national health quality and national social development and security.^3^^,^^6^ Myopia not only causes severe clinical and developmental effects but also poses a tremendous economic burden, with uncorrected myopia and myopic macular degeneration costing $244 billion and $6 billion in productivity loss, respectively.^4^ The total annual cost of myopia in urban China was $26.3 billion, accounting for 0.23% of gross domestic product (GDP).^7^

Myopia is a common eye disease in children and adolescents. Children's subjective feelings are unreliable, and they cannot accurately identify the early signs of myopia and express them correctly. Myopia in children and adolescents has a huge impact on their lifelong development. Therefore, it is necessary and urgent to take action against myopia in schoolchildren. The World Health Organization has listed child blindness as a disease that needs priority prevention, intervention and rehabilitation.

A large number of studies have evaluated the clinical effectiveness of health education, vision screening, outdoor activities and various intervention methods in the field of myopia prevention and control, and digital medicine is also playing an increasingly important role in myopia prevention and control.^8^, ^9^, ^10^, ^11^, ^12^ Various digital healthcare technologies have been widely used in school-based screening covering millions of children and adolescents, health education, behavior change and risk warning. Successful pilot projects also provide a further evaluation of their effects.

However, the role of a single intervention method is limited, and a comprehensive myopia prevention and control program can maximize the effects of each part. Integrated management implies more investment, and therefore requires a comprehensive economic assessment at the national level.^3^ Nevertheless, there is a lack of cost-effectiveness analysis of comprehensive myopia prevention and control programs, and the only studies have focused on school-based myopia screening.^12^, ^13^, ^14^

To fill this huge gap, we compared the cost-utility and cost-effectiveness between a myopia prevention and control strategy with or without digital technology and single school-based myopia screening program for schoolchildren aged 6–18 years old in rural and urban China from a societal perspective, which could provide information for realizing a high-quality, affordable and sustainable myopia prevention and management plan for China and other countries.

## Methods

### Overview of the Markov model

We conducted a Markov model by TreeAge Pro (TreeAge Software; Williamstown, MA, USA) to compare the cost-utility and cost-effectiveness of traditional myopia prevention and control strategy, digital comprehensive myopia prevention and control strategy, and school-based vision test mode among 100,000 schoolchildren in rural and urban China (Appendix Fig. S1). This model simulated the natural progression of myopia in a hypothetical cohort of 6-year-old schoolchildren until they reached the age of 18. The model consisted of 4 stages according to cycloplegic spherical equivalent (SE): non-myopia (SE > −0.5 diopters [D]), low myopia (−3.00 D < SE ≤ −0.50 D), moderate myopia (−6.00 D < SE ≤ −3.00 D), and high myopia (SE ≤ −6.00 D). Seen in Appendix Fig. S2. Primary parameters were derived from real-world studies, meta-analysis, pilot projects, official data published by the National Health Commission of the People's Republic of China and the National Expert Advisory Committee on Vision Health Management for Children and Adolescents, and reasonable assumptions.

### Overview of myopia management strategies in China

School-based myopia screening strategy: Routine vision tests were carried out in primary and secondary schools every semester, with a screening coverage rate of 91.8% (public data of National Health Commission of the People's Republic of China, http://www.nhc.gov.cn/). Students who initially screened positive were recommended to the local optometry centers or hospitals for detailed cycloplegic examination and subsequent treatment under the guidance of their parents.

Traditional myopia prevention and control strategy: The Chinese government launched the Implementation Plan for the Comprehensive Prevention and Control of Myopia among Children and Adolescents in 2018, emphasizing the important roles of health education in the prevention and control of myopia. Guided by the government and local hospitals, schools undertook the work of myopia-related health education for students and parents. Commonly used publicity channels included posting posters, playing videos, distributing brochures, carrying out thematic activities, and encouraging students to take part in more outdoor activities.^15^

Digital comprehensive myopia prevention and control strategy: Unlike the traditional myopia management strategy, short messages and popular science articles were sent through novel digital health education channels like WeChat and SMS.^10^^,^^16^

All three myopia management strategies included school-based vision tests. In China, school-based vision tests usually used non-cycloplegic measurements, which resulted in specific specificity and sensitivity. For both traditional and digital myopia prevention and control strategies, students who initially screened positive were recommended to the local optometry centers or hospitals, where they received a comprehensive eye examination (including cycloplegic examination), treatment and follow-up. Medical institutions transmitted the test results back to the school, which was responsible for updating the electronic records.

### Primary parameters in the Markov model

#### Cost

Under the guidance of the National Expert Advisory Committee on Vision Health Management for Children and Adolescents, annual costs for health education, school screening, hospital diagnostic examination and treatment were ascertained according to current school myopia screening programs in China.^7^^,^^14^^,^^17^^,^^18^ Since we used a societal perspective, both direct and indirect costs were taken into consideration. Direct costs consisted of costs created inside and outside the hospital for myopic correction and follow-up examinations, as well as costs related to transportation, food, and accommodation associated with the visits; indirect costs included family members’ working time loss and accompanying costs due to myopia treatments.^7^ Both capital and recurrent costs were included in our study. Screening and health education costs were recorded in Chinese yuan and converted into US dollars at the average exchange rate in 2021 (one dollar was equal to 6.45 yuan). Data for the intervention costs in 2021 were adjusted from previous data at an annual interest rate of 5%.^17^ The composition of costs is shown in the Appendix Tables S6–S9.

#### Main outcomes

Both cost-utility and cost-effectiveness analyses were conducted. Incremental cost-utility ratios (ICURs) were calculated using the following formula: ICURs=(incrementalcost)/(qualityadjustedlifeyearsgained). Incremental cost-effectiveness ratios (ICERs) were calculated using the following formula: ICERs=(incrementalcost)/(disabilityadjustedlifeyearsaverted). In terms of the cost-effectiveness threshold, WHO defined interventions with a cost lower than the per capita GDP as highly cost-effective, those with a cost of one to three times the per capita GDP as cost-effective, and those with a cost higher than three times the per capita GDP as non-cost-effective.^19^ According to the overall per capita national GDP ($12,551), urbanization rate (0.65), and urban‒rural ratio (2.5) of per capita disposable income, the per capita GDPs for rural and urban settings were $10,167 and $13,856 in 2021, respectively (Appendix Table S10). The cost-effectiveness thresholds were $30,501 and $41,568, respectively, per quality-adjusted life-year (QALY) gained or per disability-adjusted life-year (DALY) averted for both settings.^19^

### Sensitivity analyses

Broad sensitivity analyses were conducted to reflect the uncertainty and robustness of base-case scenarios. A floating range of 10% or 20% of basic values was used as the upper and lower bounds of the one-way sensitivity analysis.^20^ For probabilistic sensitivity analysis (PSA), we reassigned β or γ distributions for parameters and recalculated ICURs for 10,000 Monte Carlo simulations.^20^^,^^21^ Subgroup sensitivity analyses were performed by changing the parameters for implementing network health education. The 2.5th and 97.5th percentiles of costs, QALYs, and DALYs were calculated using methods mentioned in previous studies.^22^

### Role of the funding source

The funder of the study had no role in the study design, data collection, data analysis, data interpretation, or writing of the report.

## Results

**Fig. 1:**
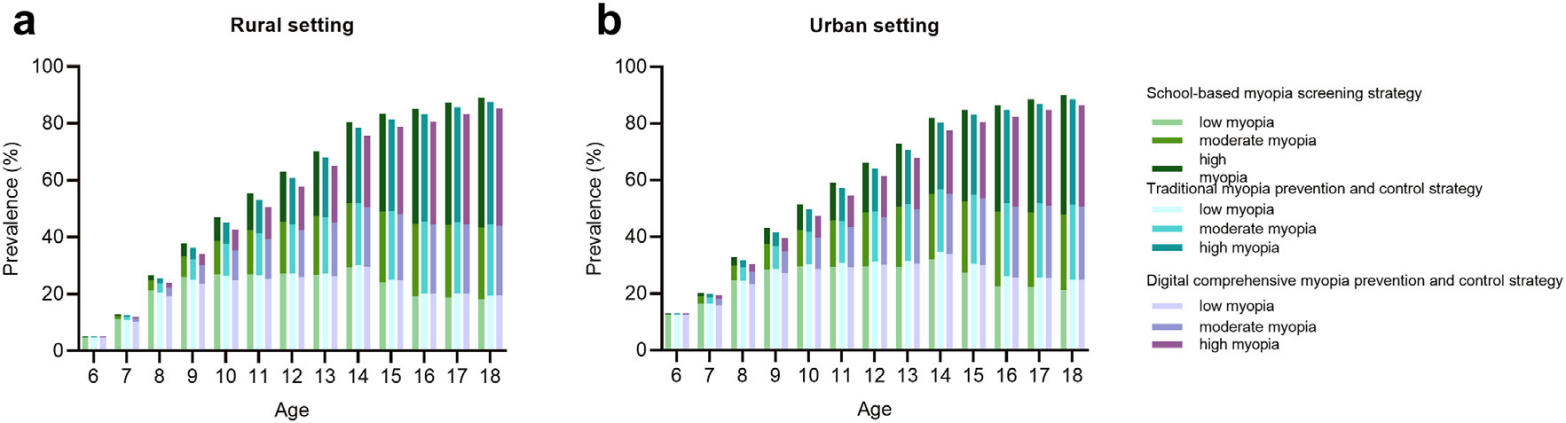
Temporal trends of myopia prevalence from 6 to 18-year-old schoolchildren. This figure shows the prevalence of myopia among students aged 6-18 and the trend of the prevalence of low, moderate and high myopia. **a**. The baseline prevalence rates of low, moderate and high myopia were 4.83%, 0.07%, and 0.09%, among rural 6-year-old children. The prevalence of myopia among 18-year-old rural students was 89.16%, 87.63% and 85.37% under school-based myopia screening strategy, traditional myopia prevention and control strategy and digital comprehensive myopia prevention and control strategy. **b**. The baseline prevalence rates of low, moderate and high myopia were 12.56%, 0.19%, and 0.25% among urban 6-year-old children. The prevalence of myopia among 18-year-old urban students was 90.04%, 88.64% and 86.56% under school-based myopia screening strategy, traditional myopia prevention and control strategy and digital comprehensive myopia prevention and control strategy.

The baseline prevalence rates of low, moderate and high myopia were 4.83%, 0.07%, and 0.09%, respectively, among rural 6-year-old children and 12.56%, 0.19%, and 0.25%, respectively, among urban 6-year-old children. Under the current scenario of school-based myopia screening strategy, the prevalence of myopia among 18-year-old students in rural and urban areas was 89.16% and 90.04% at the end of the Markov cycles, respectively. The prevalence of myopia was reduced to 87.63% and 85.37% after implementing the traditional and digital myopia prevention and control strategies in rural settings, respectively, and the figures were 88.64% and 86.56%, respectively, in urban settings. Additionally, compared with school-based myopia screening program, the prevalence of high myopia decreased by 1.81% and 1.35% among rural and urban students in digital myopia prevention and control strategy (Fig. 1).

The base-case cost-utility comparison among the school-based myopia screening strategy, traditional myopia prevention and control strategy and digital comprehensive myopia prevention and control strategy is presented in Table 1. The cumulative costs incurred and QALYs gained by a 6-year-old schoolchild in a rural setting in previous practice were $171 (137–206) and 9.57764 (9.57524–9.58036), and $607 (488–729) and 9.56321 (9.55973–9.56668) in an urban setting. In rural settings, the incremental cost per QALY gained with the traditional ($6309) and digital myopia prevention and control strategies ($11,301) both satisfied the cost-effectiveness threshold ($30,501), and the digital myopia management strategy yielded the largest health benefits (9.59448 QALYs per person). In urban settings, the ICUR produced by the digital myopia management plan ($10,707) was less than 1 times the per capita GDP ($13,856), which implied that implementing a national-level digital comprehensive myopia prevention and control strategy was highly cost-effective in urban areas and cost-effective in rural areas. However, in cost-effectiveness analysis, the ICERs produced by digital comprehensive myopia prevention and control strategy ($37,446 per DALY averted in rural setting, $41,814 per DALY averted in urban setting) slightly exceeded the cost-effectiveness threshold in both rural and urban settings.

**Table 1:** Base-case results of cost-utility analysis.

	Costs per person (2.5th and 97.5th percentiles), $	QALYs per person (2.5th and 97.5th percentiles)	Incremental costs per 100,000 people, $	Incremental QALYs per 100,000 people	ICURs, $	DALYs per person (2.5th and 97.5th percentiles)	Incremental DALYs averted per 100,000 people	ICERs, $	Comparison strategy for ICUR and ICER calculation^a^
Rural setting									
School-based myopia screening strategy	171 (137–206)	9.57764 (9.57524–9.58036)	–	–	–	0.06562 (0.06022–0.07132)	–	–	–
Traditional myopia prevention and control strategy	218 (178–262)	9.58509 (9.58024–9.59102)	4,700,000	745	6,309	0.06189 (0.05658–0.06738)	373	**12,588**	School-based myopia screening strategy
Digital comprehensive myopia prevention and control strategy	324 (279–374)	9.59448 (9.58586–9.60535)	10,600,000	939	**11,301**	0.05905 (0.05342–0.06472)	284	37,446	Traditional myopia prevention and control strategy
Urban setting									
School-based myopia screening strategy	607 (488–729)	9.56321 (9.55973–9.56668)	–	–	–	0.06552 (0.05989–0.07176)	–	–	–
Traditional myopia prevention and control strategy	729 (583–876)	9.57119 (9.56590–9.57719)	12,200,000	798	15,271	0.05886 (0.05324–0.06514)	666	**18,295**	School-based myopia screening strategy
Digital comprehensive myopia prevention and control strategy	819 (676–966)	9.57964 (9.57129–9.58974)	9,000,000	845	**10,707**	0.05670 (0.05100–0.06303)	216	41,814	Traditional myopia prevention and control strategy

QALY = quality-adjusted life-year. ICUR = incremental cost-utility ratio. DALY = disability-adjusted life-year. ICER = incremental cost-effectiveness ratio. Costs are given in US dollars. Both direct and indirect costs are taken into consideration. The cost-effectiveness thresholds are $30,501 per QALY gained or per DALY averted for rural settings and $41,568 per QALY gained or per DALY averted for urban settings. Costs, QALYs, and DALYs are defined as lifetime values (from 6 to 18 years old) per person, whereas incremental costs, incremental QALYs, ICURs, incremental DALYs averted, and ICERs are defined as values per 100,000 people.

a Extended dominance analysis is conducted, all screening methods are compared to the previous screening method.

To test how digital myopia prevention and control strategy can be optimally cost-effectiveness, subgroup analyses were conducted. We assumed a 100% improvement in compliance with school-based vision tests, outdoor activities, hospital examinations or interventions in digital myopia management strategy (Table 2). In both rural and urban areas, full coverage of outdoor activities ($24,197 and $24,417 per DALY averted in rural and urban settings, respectively) and spectacles wearing ($26,399 and $20,676 per DALY averted in rural and urban setting, respectively) satisfied the cost-effectiveness threshold. Full coverage of outdoor activities generated the lowest cumulative cost ($321 for rural settings and $808 for urban settings).

**Table 2:** Subgroup cost-effectiveness analyses for digital comprehensive myopia prevention and control strategies with perfect compliance.

	Base-case value	Cost per person, $	DALYs per person	Incremental costs per person, $	Incremental DALYs averted per person	ICERs, $	Comparison strategy for ICER calculation^a^
Rural setting							
Traditional myopia prevention and control strategy	–	218	0.06189	–	–	–	–
Perfect compliance with outdoor activities	83.8%	321	0.05766	102	0.00423	**24,197**	Traditional myopia prevention and control strategy
Perfect compliance with full coverage of school-based vision test	91.8%	343	0.05878	22	−0.00112	−19,891	Perfect compliance with outdoor activities
Perfect compliance with spectacles	36%	484	0.05342	142	0.00536	**26,399**	Perfect compliance with full coverage of school-based vision test
Perfect compliance with full hospital examination	35.3%	683	0.05324	199	0.00018	1,103,456	Perfect compliance with spectacles
Urban setting							
Traditional myopia prevention and control strategy	–	729	0.05886	–	–	–	–
Perfect compliance with outdoor activities	83.8%	808	0.05564	79	0.00323	**24,417**	Traditional myopia prevention and control strategy
Perfect compliance with full coverage of school-based vision test	91.8%	882	0.05534	74	0.00029	253,093	Perfect compliance with outdoor activities
Perfect compliance with spectacles	68.3%	1,003	0.04949	121	0.00586	**20,676**	Perfect compliance with full coverage of school-based vision test
Perfect compliance with full hospital examination	73.3%	1,071	0.05107	68	−0.00158	−42,612	Perfect compliance with spectacles

DALY = disability-adjusted life-year. ICER = incremental cost-effectiveness ratio. Costs are given in US dollars. Both direct and indirect costs are taken into consideration. ICERs are recalculated by increasing the base-case values to 100%. The cost-effectiveness thresholds are $30,501 per DALY averted for rural settings and $41,568 per DALY averted for urban settings. Costs, DALYs, incremental costs, incremental DALYs averted, and ICERs are defined as lifetime values (from 6 to 18 years old) per person.

a Extended dominance analysis is conducted, all screening methods are compared to the previous screening method.

Broad sensitivity analyses were conducted. In the one-way sensitivity analysis, the base-case results were insensitive to a wide range of parameter fluctuations in traditional and digital myopia prevention and control strategies in both rural and urban settings, with ICURs within 3 times the per capita GDP (Appendix Fig. S5). PSA showed that the traditional and digital comprehensive strategy had a 100% and 99.72% probability of being cost-effective at the threshold of 3 times the per capita GDP for rural settings, and the figures for urban settings were 99.89% and 99.96%, respectively (Appendix Fig. S6). The acceptability curve indicated that the digital myopia prevention and control strategy was the most cost-effective option at the current cost-effectiveness threshold, with a probability of 99.72% in rural areas and 99.94% in urban areas (Appendix Fig. S7).

## Discussion

To the best of our knowledge, this is the first health economic study to compare the cost-effectiveness among traditional school-based myopia screening programs and comprehensive myopia prevention and control strategies in China. Overall, our results showed that implementing a digital comprehensive myopia prevention and control strategy for children and adolescents in China is the most cost-effective option compared with traditional school-based myopia control programs.

Numerous epidemiological studies have confirmed the early onset age, high degree and rapid progression of myopia in children and adolescents.^23^^,^^24^ Preschool and early-school age are critical periods for the onset and development of myopia, and a standardized screening program during this period can help us to understand the prevalence of myopia and implement targeted treatment and prevention measures. School-based myopia screening programs have been widely demonstrated to meet cost-effectiveness thresholds in both developed and developing countries.^13^^,^^14^^,^^25^ Evaluations including Africa, Asia, the Americas, and Europe confirmed annual screening for the 11-15-year-old age group as the most cost-effective intervention (incremental costs per disability-adjusted life-year, $67–$458).^13^ Another study conducted in China proved that the teacher screening strategy was the most cost-effective choice for children aged 4–5 in rural China, with a 40% lower cost per case.^14^ However, these studies have several common limitations. On the one hand, these one-off health economics assessments do not take into account that myopia is a dynamic development process; on the other hand, referral, treatment, and follow-up were not included in the model, thus producing an incomplete evaluation, and the large-scale implementation of the project requires full consideration of medical human resources, financial investment, and data management.^3^ Therefore, there is an urgent need for a better and more comprehensive cost-effectiveness assessment of myopia prevention and control programs within a broader context. Our study provides an unprecedented cost-effectiveness analysis of a comprehensive myopia management strategy that integrates screening, prevention, and treatment simultaneously.

Socioeconomic development and urbanization have contributed to the emergence of myopia in children and adolescents as a major public health problem. Consistent with previous studies, our prediction model showed that without intervention, the prevalence of myopia rates among rural and urban high school graduates was as high as 89.16% and 90.04%, respectively.^26^ The global myopia pandemic means an increased demand for testing and treatment (including spectacles, refractive surgery and other intervention methods), placing significant pressure on eye care resources and healthcare systems. Second, treatable complications, such as glaucoma and cataract, incur additional eye services and costs. More importantly, the increasing degree of myopia has led to an increasing number of sight-threatening and irreversible pathological outcomes, such as retinal detachment and macular degeneration among the middle-aged and elderly, and even among a certain percentage of the working-age population, which further increases productivity loss and economic burden.^3^ Therefore, delaying the progression of myopia and preventing the occurrence of high myopia is the future focus of myopia prevention and control. It is worth noting that a large number of clinical studies have preliminarily tested several factors affecting the progression of myopia, especially the influence of outdoor activities and near work on myopia, which has been proven by evidence-based medicine.^16^^,^^23^ Therefore, increasing outdoor activities and reducing near work have been integrated into public health policies in many countries, and have been widely promoted, including in China. In response to these two factors, the Chinese government has introduced a "one increase and one reduction" policy (increasing outdoor activities and reducing the heavy burden of study for primary and middle school students).^27^ After the practice in pilot areas, China has accumulated abundant data. We used these real-world data to further verify that more outdoor activity with full coverage produced the lowest expected cost ($321 and $808 per rural and urban student), met the cost-effectiveness threshold ($24,197 and $24,417 per DALY averted in rural and urban settings, respectively). The detailed results confirmed that full coverage of outdoor activities was one of the suitable choices for China's national conditions, and should be promoted on a large-scale.

Long-term, intensive and active myopic intervention for myopia can have a maximizing effect. However, children and adolescents are generally characterized by insufficient attention to myopia and its pathological outcomes, failure to detect early identifying symptoms of myopia in a timely manner, and insufficient motivation to actively intervene, which reduces the effect of myopia prevention and treatment.^16^ Therefore, parents play an important role in this process, but many of them also suffer from a lack of understanding and attention. Health education can improve knowledge by providing correct vision information. The World Health Organization and International Agency for the Prevention of Blindness have proposed that increasing public education was one of the primary myopia control strategy recommendations.^16^^,^^28^ Since the 1980s, Singapore and Japan have introduced health education-based policies for the prevention and control of myopia.^29^ However, traditional health education is faced with common defects, such as insufficient coverage, poor sustainability, and large manpower input in China. Therefore, the Chinese government optimized the delivery of health education according to local conditions and took the lead in using WeChat and SMS as the carriers of health education.^10^^,^^16^^,^^30^ This novel digital health education mode penetrates the daily lives of students and parents in a short, clear, intensive, operable, low-cost, and fragmented way, and it could enable immediate intervention and build awareness in a positive and sustained manner.^10^^,^^16^^,^^30^ The results of randomized controlled trials confirmed that the compliance with digital-based health education was significantly improved compared with traditional methods.^10^^,^^16^^,^^30^ Our cost-utility analyses proved the economic attraction of this digital mode in both rural and urban settings in China, and supplemented the economic data.

Like other eye diseases, myopia has faced the dilemma of increasing patients and insufficient and unevenly distributed ophthalmic human resources. The wide application of digital medicine in the field of ophthalmology has effectively improved this situation, which widely covers different regions and populations, making myopia control available, accessible and affordable. Furthermore, digital medicine plays an additional and unique role in myopia management. On the one head, a web-based program strengthens the horizontal connections between all social parties, reduces personnel, time and cost consumption, and improves utilization of services; on the other hand, digital management modes and online refractive development archives facilitate automated and vertical accumulation, evidence-based updating, dynamic analysis and risk warning of large-scale data to enable personalized myopic treatment and management, and the new myopia prevention and control strategy combining Internet of Things and blockchain technology will further increase the security, portability and transparency of data transmission and sharing.^11^^,^^31^^,^^32^ For example, China's digital school-based myopia management pilot program which covers millions of students, realizes automated information collection, intelligent analysis and online access, and reduces labor and time costs by 90% compared with traditional screening.^31^ We also concluded that implementing network-based myopia management strategies in both urban and rural areas was economically attractive, which facilitates the promotion of this modern, practical and low-cost paradigm in China. In the future, it is necessary to evaluate the most cost-effective screening strategy for different common eye diseases in different stages across the whole life cycle.

**Fig. 2:**
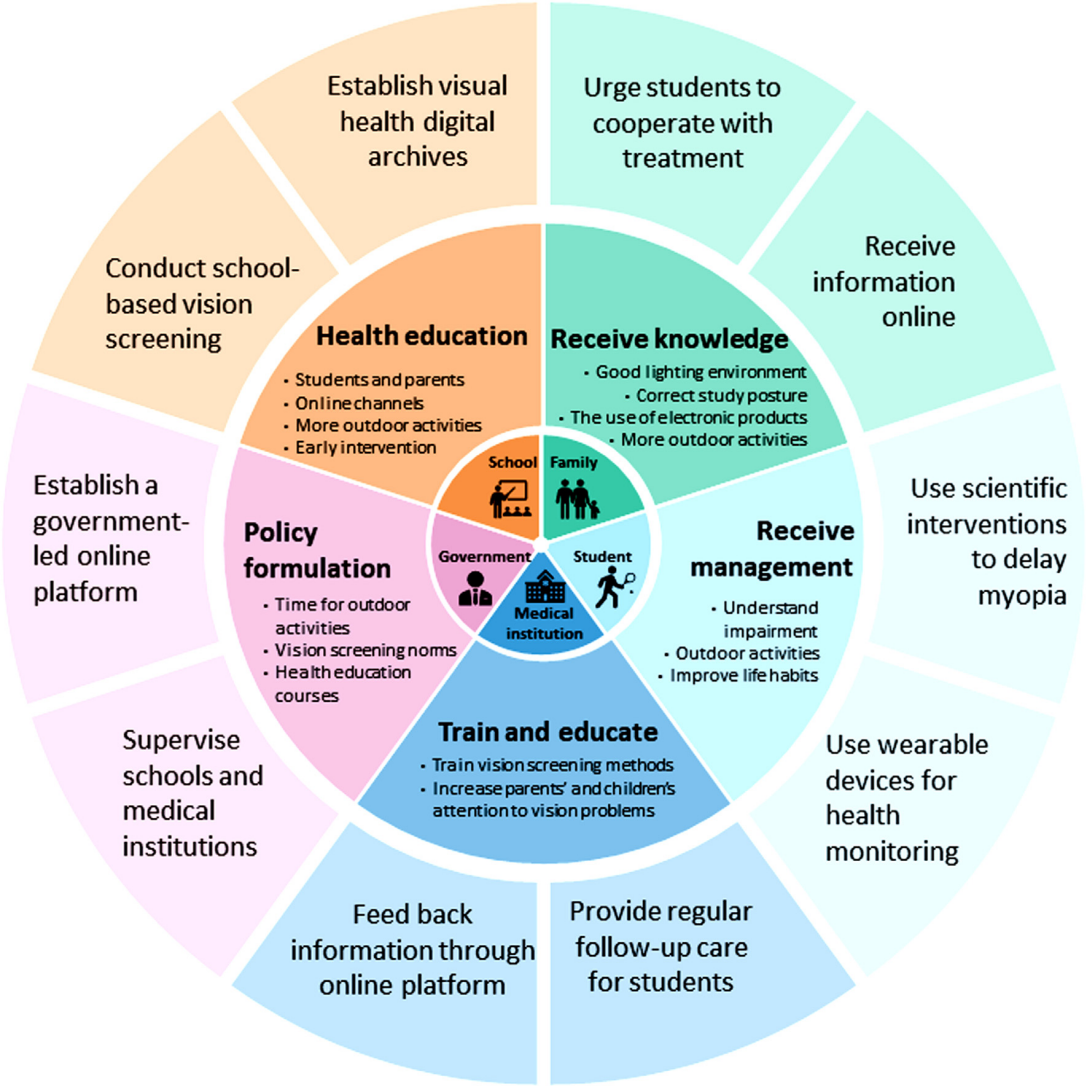
The digital comprehensive myopia prevention and control strategy including multi-party cooperation. The changing health needs of the population bring challenges to the prevention and control of myopia. In the future, myopia prevention and control for youths should be effectively integrated into general health services. Through the five-party collaboration of the government, schools, medical institutions, families and students, effective control of myopia can be achieved. As the main front of myopia prevention and control, schools should play an important role in health education. Schools and education departments need to further reform the existing education model, change the teaching environment, integrate vision education into general school education, and reduce the burden in the early years of learning. Parents should cooperate with the school, improve myopia-related knowledge, ensure that children have enough outdoor activities, and undertake after-school supervision. As beneficiaries of myopia prevention and control programs, students should be deeply aware of the serious impact of myopia on personal development. They should try their best to cooperate with the regulations of schools, hospitals and parents and use scientific interventions to reduce the incidence and development of myopia.

Under the leadership of the Chinese government, a series of pilot programs around comprehensive myopia prevention and control have been conducted, and a large number of pragmatic research-based approaches have contributed to the accumulation of evidence.^10^^,^^15^^,^^16^^,^^26^^,^^31^^,^^33^^,^^34^ Our study evaluated the cost-effectiveness of a practical, constructive and highly feasible comprehensive myopia prevention and treatment framework based on these findings. However, achieving the large-scale rollout of a comprehensive digital myopia prevention and control program still faces significant challenges. First, the implementation of a comprehensive myopia control program is a multifaceted and continuous process, and there should be no shortcomings in each link.^35^ The medical sector needs to work with schools, families and other government departments to achieve significant and tangible cobenefits (Fig. 2). Second, current strategies do not fundamentally change the distal drivers of myopia. There is an urgent need to restructure the entire educational environment at the upstream level. The reform of China's education system eases the pressure on education, especially on younger children and key schools.^10^^,^^27^^,^^36^ Third, urbanization has promoted the development of myopia in rural areas. It is necessary to improve the coverage and accessibility of medical services, strengthen health education to eliminate the social stigma of visual impairment, and realize the fairness and affordability of medical services through medical insurance projects.^15^

Our study had several limitations. First, there is currently a lack of data on the digital health education model, so we used same parameters as traditional mode, which may be the reason why ICERs exceeded cost-effectiveness threshold. By changing the parameters through subgroup analysis, we found that the digital myopia management strategy became cost-effective after improving compliance with outdoor activities and spectacles wearing. Second, although we constructed a dynamic function for myopia transition that took into account age, intervention, and disease severity (stage), making this study more consistent with clinical facts, we did not consider the effects of the natural environment between rural and urban settings (such as greening and light exposure) on myopia progression. Future modeling studies should focus more on a comprehensive consideration of the combined effects of natural and social factors on the progression of myopia. Third, routine eye disease screening programs can simultaneously monitor many common eye diseases, but the management of other common eye diseases in children, such as strabismus and various congenital eye diseases, was not incorporated into our model, leading to a potential underestimation of both costs and QALYs of the comprehensive prevention and control strategy, which should be included in future studies. Forth, given that this study focused primarily on children and adolescents aged 6–18 years, myopia-related complications such as the pathological myopia stage were not included in our study.

Before implementing major public health measures, it is necessary to carefully evaluate them to ensure the maximum effect of the measures. Our findings indicate that implementing a comprehensive digital myopia prevention and control strategy in China is cost-effective, which provides a powerful economic reference for policy-makers to promote this framework in a broader context.

## Contributors

Conception and design: Hanruo Liu, Ningli Wang and Zi-Bing Jin.

Acquisition or interpretation of data: Ruyue Li, Kaiwen Zhang, Zhecheng Lu, Yue Zhang, Huiqi Li, Jiaxin Tian, Shi-Ming Li, Li Li, Liyuan Wang, Xiuhua Wan, Fengju Zhang.

Drafting of the manuscript: Ruyue Li.

Critical revision of the manuscript for important intellectual content: Hanruo Liu and Ningli Wang.

Statistical analysis: Ruyue Li.

Obtained funding: Hanruo Liu, Li Li, Shi-Ming Li.

Supervision: Hanruo Liu and Ningli Wang accessed and verified the data. Hanruo Liu and Ningli Wang were responsible for the decision to submit the manuscript.

## Data sharing statement

The parameters that we used in our model (text, tables, figures, models, and appendices) are available on reasonable request from the corresponding author (Hanruo Liu; hanruo.liu@hotmail.co.uk) under certain conditions (with the consent of all participating centers and with a signed data access agreement).

## Declaration of interests

The authors declare no conflicts of interest.

## Appendix A Supplementary data

Translated abstract

## References

[1] Holden B.A., Fricke T.R., Wilson D.A. (2016). Global prevalence of myopia and high myopia and temporal trends from 2000 through 2050. Ophthalmology.

[2] Sankaridurg P., Tahhan N., Kandel H. (2021). IMI impact of myopia. Invest Ophthalmol Vis Sci.

[3] Burton M.J., Ramke J., Marques A.P. (2021). The lancet global health commission on global eye health: vision beyond 2020. Lancet Glob Health.

[4] Modjtahedi B.S., Abbott R.L., Fong D.S., Lum F., Tan D. (2021). Reducing the global burden of myopia by delaying the onset of myopia and reducing myopic progression in children: the academy's task force on myopia. Ophthalmology.

[5] Bullimore M.A., Ritchey E.R., Shah S., Leveziel N., Bourne R.R.A., Flitcroft D.I. (2021). The risks and benefits of myopia control. Ophthalmology.

[6] Pan C.-W., Wu R.K., Wang P., Li J., Zhong H. (2018). Reduced vision, refractive errors and health-related quality of life among adolescents in rural China. Clin Exp Optom.

[7] Ma Y., Wen Y., Zhong H. (2022). Healthcare utilization and economic burden of myopia in urban China: a nationwide cost-of-illness study. J Glob Health.

[8] Wei S., Li S.-M., An W. (2020). Safety and efficacy of low-dose atropine eyedrops for the treatment of myopia progression in Chinese children: a randomized clinical trial. JAMA Ophthalmol.

[9] Wolffsohn J.S., Flitcroft D.I., Gifford K.L. (2019). IMI - myopia control reports overview and introduction. Invest Ophthalmol Vis Sci.

[10] Li S.M., Ran A.-R., Kang M.T. (2022). Effect of text messaging parents of school-aged children on outdoor time to control myopia: a randomized clinical trial. JAMA Pediatr.

[11] Keel S., Govender-Poonsamy P., Cieza A. (2022). The WHO-itu MyopiaEd programme: a digital message programme targeting education on myopia and its prevention. Front Public Health.

[12] Griffiths U.K., Bozzani F.M., Gheorghe A., Mwenge L., Gilbert C. (2014). Cost-effectiveness of eye care services in Zambia. Cost Eff Resour Allocation.

[13] Baltussen R., Naus J., Limburg H. (2009). Cost-effectiveness of screening and correcting refractive errors in school children in Africa, Asia, America and Europe. Health Policy.

[14] Wang L., Congdon N., Hogg R.E. (2019). The cost-effectiveness of alternative vision screening models among preschool children in rural China. Acta Ophthalmol.

[15] Ma X., Zhou Z., Yi H. (2014). Effect of providing free glasses on children's educational outcomes in China: cluster randomized controlled trial. BMJ.

[16] Li Q., Guo L., Zhang J. (2021). Effect of school-based family health education via social media on children's myopia and parents' awareness: a randomized clinical trial. JAMA Ophthalmol.

[17] Tang J., Liang Y., O'Neill C., Kee F., Jiang J., Congdon N. (2019). Cost-effectiveness and cost-utility of population-based glaucoma screening in China: a decision-analytic Markov model. Lancet Glob Health.

[18] Zhang Y., Guan H., Du K. (2021). Effects of vision health education and free eyeglasses on knowledge of vision and usage of spectacles among primary school students: evidence from gansu and shaanxi provinces in China. Risk Manag Healthc Policy.

[19] Hutubessy R., Chisholm D., Edejer T.T.-T. (2003). Generalized cost-effectiveness analysis for national-level priority-setting in the health sector. Cost Eff Resour Allocation.

[20] Briggs A.H. (2000). Handling uncertainty in cost-effectiveness models. Pharmacoeconomics.

[21] Hatswell A.J., Bullement A., Briggs A., Paulden M., Stevenson M.D. (2018). Probabilistic sensitivity analysis in cost-effectiveness models: determining model convergence in cohort models. Pharmacoeconomics.

[22] Polsky D., Glick H.A., Willke R., Schulman K. (1997). Confidence intervals for cost-effectiveness ratios: a comparison of four methods. Health Econ.

[23] Li S.-M., Liu L.-R., Li S.-Y. (2013). Design, methodology and baseline data of a school-based cohort study in Central China: the Anyang Childhood Eye Study. Ophthalmic Epidemiol.

[24] Jonas J.B., Ang M., Cho P. (2021). IMI prevention of myopia and its progression. Invest Ophthalmol Vis Sci.

[25] Frick K.D., Riva-Clement L., Shankar M.B. (2009). Screening for refractive error and fitting with spectacles in rural and urban India: cost-effectiveness. Ophthalmic Epidemiol.

[26] He M., Xiang F., Zeng Y. (2015). Effect of time spent outdoors at school on the development of myopia among children in China: a randomized clinical trial. JAMA.

[27] Morgan I.G., Jan C.L. (2022). China turns to school reform to control the myopia epidemic: a narrative review. Asia Pac J Ophthalmol (Phila).

[28] Ang M., Flanagan J.L., Wong C.W. (2020). Review: myopia control strategies recommendations from the 2018 WHO/IAPB/BHVI meeting on myopia. Br J Ophthalmol.

[29] Seet B., Wong T.Y., Tan D.T. (2001). Myopia in Singapore: taking a public health approach. Br J Ophthalmol.

[30] Enthoven C.A., Derks I.P.M., Polling J.R. (2021). Ecological momentary interventions-can more smartphone use result in less myopia?. JAMA Ophthalmol.

[31] Xu L., Zhuang Y., Zhang G. (2021). Design, methodology, and baseline of whole city-million scale children and adolescents myopia survey (CAMS) in Wenzhou, China. Eye Vis (Lond).

[32] Tan T.-E., Anees A., Chen C. (2021). Retinal photograph-based deep learning algorithms for myopia and a blockchain platform to facilitate artificial intelligence medical research: a retrospective multicohort study. Lancet Digit Health.

[33] Wu P.-C., Chen C.-T., Lin K.-K. (2018). Myopia prevention and outdoor light intensity in a school-based cluster randomized trial. Ophthalmology.

[34] He X., Sankaridurg P., Wang J. (2022). Time outdoors in reducing myopia: a school-based cluster randomized trial with objective monitoring of outdoor time and light intensity. Ophthalmology.

[35] Greer S.L., Falkenbach M., Siciliani L., McKee M., Wismar M., Figueras J. (2022). From health in all policies to health for all policies. Lancet Public Health.

[36] Morgan I.G. (2016). What public policies should be developed to deal with the epidemic of myopia?. Optom Vis Sci.

