# Supplementary material for: Implementing a digital comprehensive myopia prevention and control strategy for children and adolescents in China: a cost-effectiveness analysis
Source: Lancet Reg Health West Pac. 2023 Jul 13;38:100837. doi: 10.1016/j.lanwpc.2023.100837 (PMC10372367; doi:10.1016/j.lanwpc.2023.100837)
Supplement: Translated abstract [file mmc1.docx]

背景：儿童青少年近视是重要的公共卫生问题。尽管各种近视干预措施的临床效果已经得到了广泛验证，但从国家层面来讲，缺乏对近视综合防控方案的经济性和有效性的整体评估。本研究旨在比较中国传统近视防控方案、数字化近视综合防控方案和基于学校的近视筛查项目的成本效益。

方法：采用马尔可夫模型比较6-18岁城乡学龄儿童校本近视筛查、传统近视防控策略和数字化近视综合防控策略的成本-效用和成本-效果。参数主要源于已发表的文献。主要结果为质量调整生命年（QALY）、伤残调整生命年（DALY）、增量成本-效用比（ICUR）和增量成本-效果比（ICER）。通过广泛的敏感性分析检验结果的稳健性和敏感性。

结果：与校本筛查策略相比，实施数字化近视综合防控策略后，农村和城市18岁学生的近视患病率分别下降了3.79%和3.48%。在成本-效用分析中，使用数字化近视综合防控方案，农村和城市每获得的1个QALY的ICUR分别为11,301和10,707美元，低于农村地区3倍人均GDP（30,501美元），低于城市地区1倍人均GDP（13,856美元）。在成本-效果分析中，数字化近视综合防控方案产生的ICER略高于成本效益阈值，农村和城市中每避免1个DALY产生的ICER分别为37,446美元和41,814美元。在亚组分析中，分别将户外活动和佩戴眼镜的依从性提高至100%后，数字化综合防控方案产生的ICER满足成本效益阈值，其中户外活动全覆盖产生的累计成本最低（农村321美元，城市808美元）。

结论：本研究在国家层面充分证明了推广儿童青少年数字化近视综合防控方案的成本效益。为政府、决策者和其他近视高发国家采取进一步行动提供了经济学和公共卫生参考。

基金：国家自然科学基金(82171051)，北京市自然科学基金(JQ20029)，首都健康研究与发展专项(2020-2-1081)，国家自然科学基金(82071000)，国家自然科学基金(8197030562)

**This translation in Chinese was submitted by the authors and we reproduce it as supplied. It has not been peer reviewed. Our editorial processes have only been applied to the original abstract in English, which should serve as reference for this manuscript.**
