# Supplementary material for: Implementing a digital comprehensive myopia prevention and control strategy for children and adolescents in China: a cost-effectiveness analysis
Source: Lancet Reg Health West Pac. 2023 Jul 13;38:100837. doi: 10.1016/j.lanwpc.2023.100837 (PMC10372367; doi:10.1016/j.lanwpc.2023.100837)
Supplement: Supplementary Figures S1–S7 and Tables S1–S10 [file mmc2.docx]

**Content**

**Figure 1.** Markov model for included myopia prevention and control strategies

**Figure 2.** Transition mode for myopia before and after intervention

**Table 1.** The basic characteristics of included studies

**Figure 3.** Prevalence of myopic patients among urban Chinese children

**Figure 4.** Prevalence of myopic patients among rural Chinese children

**Table 2.** Prevalence of myopia and proportion of low myopia, moderate myopia and high myopia in rural and urban settings

**Table 3.** Coverage, compliance, sensitivity and specificity of different intervention methods

**Table 4.** Transition rate before and after interventions

**Table 5.** Utility, disability weight and other parameters

**Table 6**. Cost computation of school-based vision test

**Table 7**. Cost computation of traditional health education

**Table 8**. Cost computation of digital health education

**Table 9.** Cost computation of medical intervention

**Table 10.** The calculation formula of rural and urban per capita GDP

**Figure 5.** Deterministic 1-way sensitivity analysis

**Figure 6.** Results of probabilistic sensitivity analysis

**Figure 7.** Acceptability curve of school-based myopia screening strategy, traditional myopia prevention and control strategy and digital comprehensive myopia prevention and control strategy in rural and urban settings

**Figure 1.** Markov model for included myopia prevention and control strategies


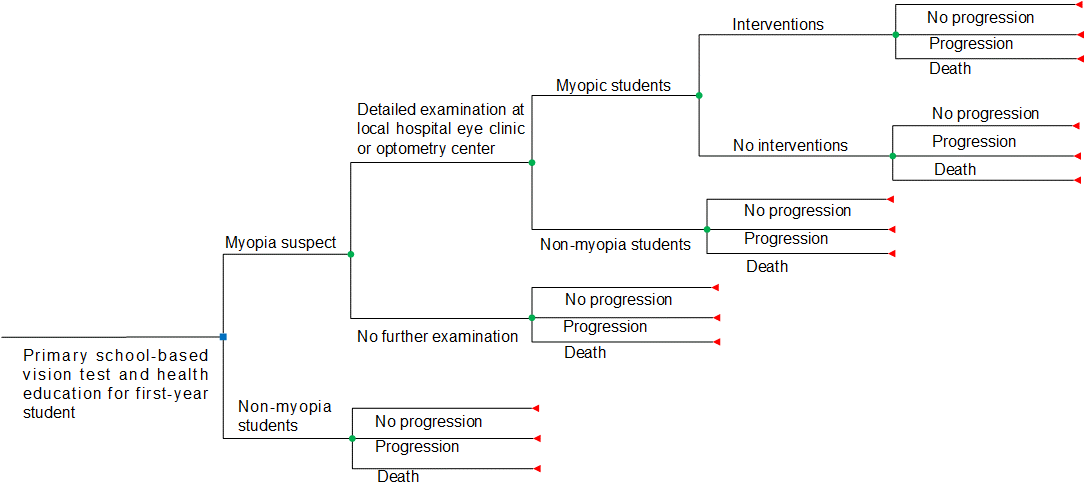


**Figure 2.** Transition mode for myopia before and after intervention


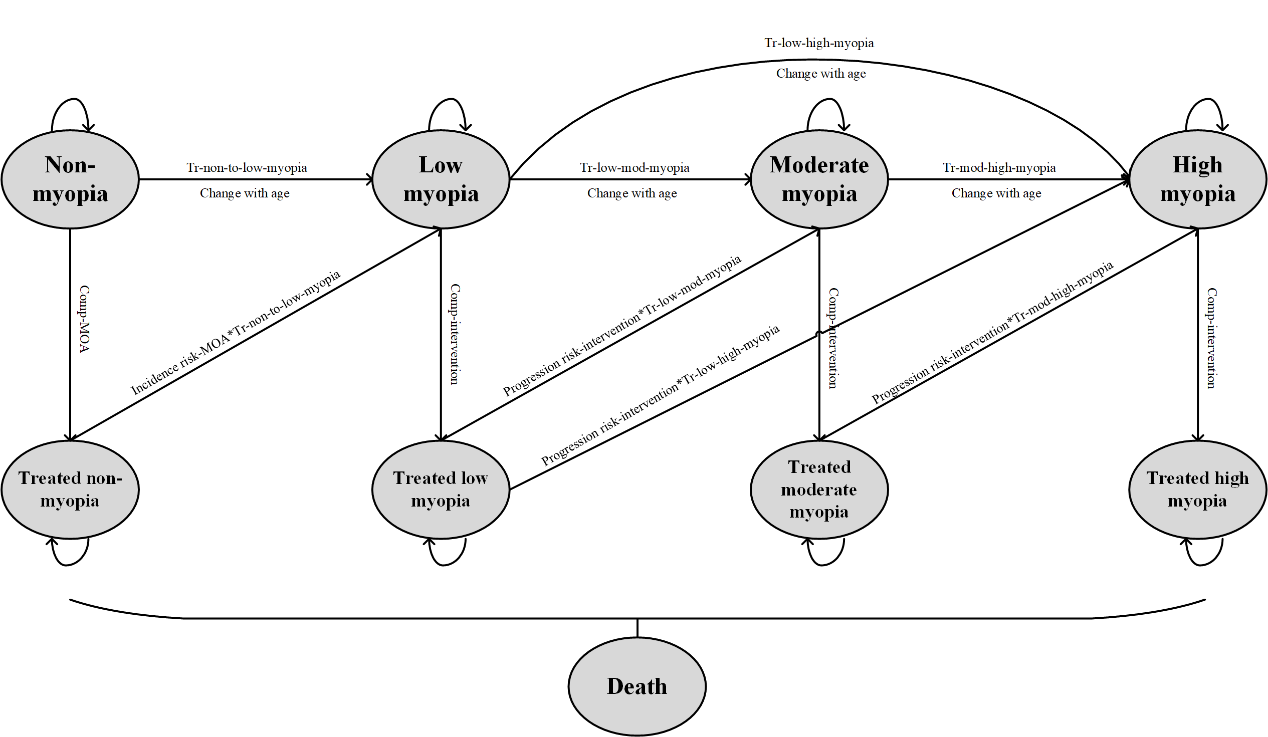


Comp=compliance. MOA=more outdoor activity. Tr=transition. Detailed parameters are seen in **Appendix Table 2-5**.

We reviewed the published literature related to the prevalence of myopia in Chinese children up to May 9, 2023 in three databases, Pubmed, EMBASE, and Web of Science, with the following search formula: "China" OR "Chinese" OR "Taiwan" OR "Macau" OR "Macao" OR "Hong Kong" AND "refractive errors" OR " myopia" OR "hyperopia" OR "astigmatism" AND Prevalence" OR "Epidemiology " OR "epidemiology" OR "prevalence" OR "incidence". We identified 18 and 9 studies to estimate the prevalence of myopia in urban and rural Chinese children, and the results of the meta-analysis showed prevalence rates of 13% and 5%, respectively.

**Table 1.** The basic characteristics of included studies

| **Author** | **Study year** | **Region (rural or urban)** | **Sample size** | **Age (mean or range)** | **Girls (%)** | **Refraction type** | **Cycloplegia (yes or no)** |
| --- | --- | --- | --- | --- | --- | --- | --- |
| He^1^ | 2008 | U | 295 | 6 | NA | AU+RE | Y |
| Liu^2^ | 2022 | U | 108 | 6 | NA | AU+RE | Y |
| Zhang^3^ | 2015 | U | 307 | 6 | NA | AU | Y |
| Zhang^3^ | 2016 | U | 559 | 6 | NA | AU | Y |
| Zhang^3^ | 2017 | U | 438 | 6 | NA | AU | Y |
| Zhang^3^ | 2018 | U | 2485 | 6 | NA | AU | Y |
| Zhang^3^ | 2019 | U | 3090 | 6 | NA | AU | Y |
| Zhang^3^ | 2020 | U | 400 | 6 | NA | AU | Y |
| Zhang^3^ | 2021 | U | 718 | 6 | NA | AU | Y |
| Zhang^4^ | 2021 | U | 224 | 6 | NA | AU | Y |
| Yang^5^ | 2021 | U | 327 | 6 | NA | AU | Y |
| Li^6^ | 2022 | U | 570 | 6 | NA | AU | Y |
| Hsieh^7^ | 2022 | U | 402 | 6 | 64% | AU | Y |
| Leng^8^ | 2021 | U | 1510 | 6 | NA | AU | Y |
| Mu^9^ | 2023 | U | 2409 | 6 | 46% | AU+RE | Y |
| Lin^10^ | 2023 | U | 3304 | 6 | NA | AU | Y |
| Yue^11^ | 2022 | U | 817 | 6 | NA | AU | Y |
| Ye^12^ | 2022 | U | 811 | 6 | NA | AU | Y |
| Ma^13^ | 2016 | R | 862 | 6 | NA | AU | Y |
| Ma^13^ | 2016 | R | 853 | 7 | NA | AU | Y |
| Li^14^ | 2014 | R | 436 | 5-9 | NA | AU | Y |
| Pan^15^ | 2018 | R | 1089 | 7.7 | 45% | AU | Y |
| Pi^16^ | 2010 | R | 239 | 6 | 33% | RE | Y |
| Pi^16^ | 2010 | R | 313 | 7 | 46% | RE | Y |
| Pi^16^ | 2010 | R | 339 | 8 | 54% | RE | Y |
| Xia^17^ | 2010 | R | 793 | 7 | NA | AU | Y |
| Xia^17^ | 2010 | R | 737 | 8 | NA | AU | Y |

U=urban. R=rural. AU=auto refraction. RE= retinoscopy. Y=yes.

**Figure 3.** Prevalence of myopic patients among urban Chinese children

**
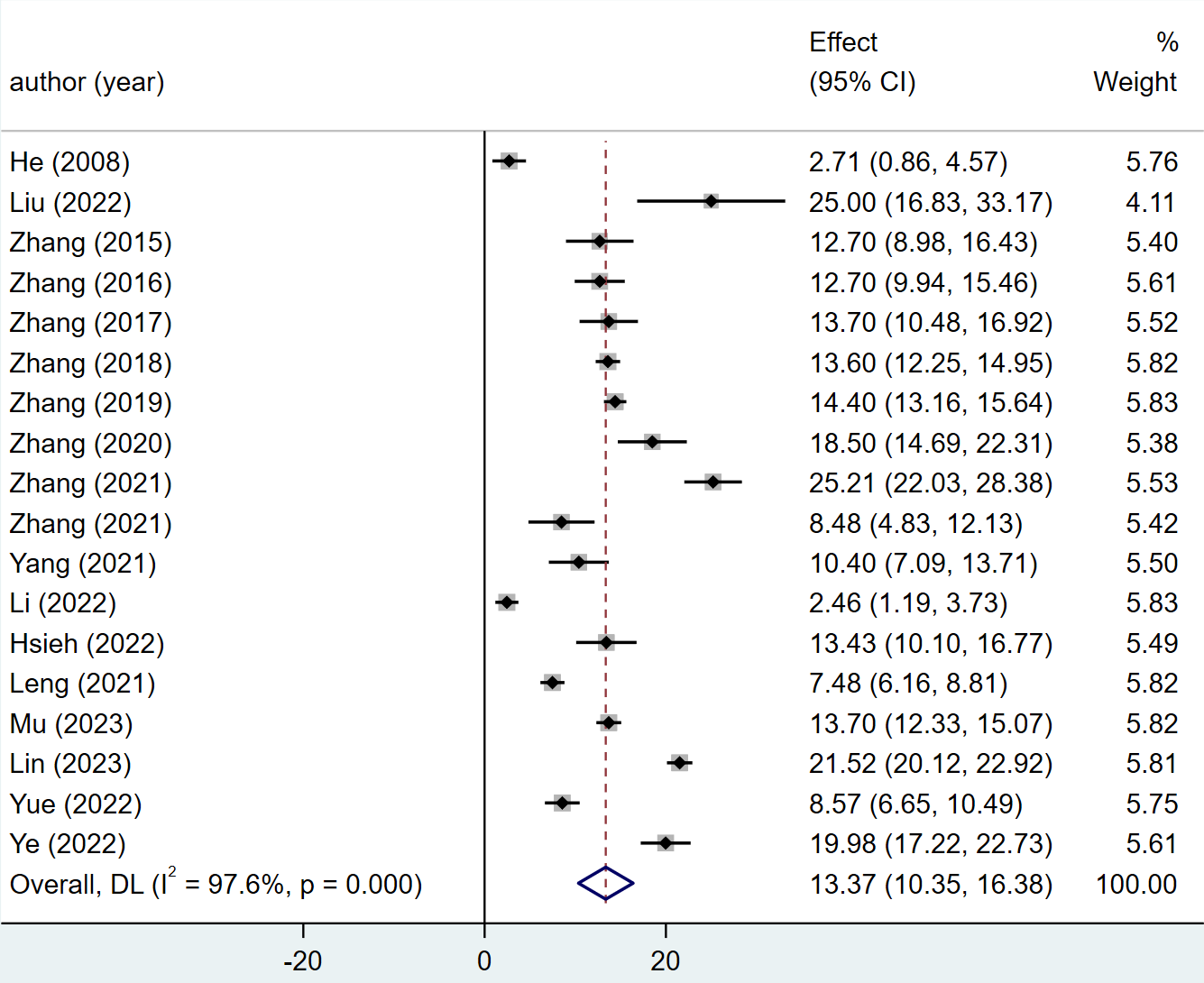
**

**Figure 4.** Prevalence of myopic patients among rural Chinese children

**
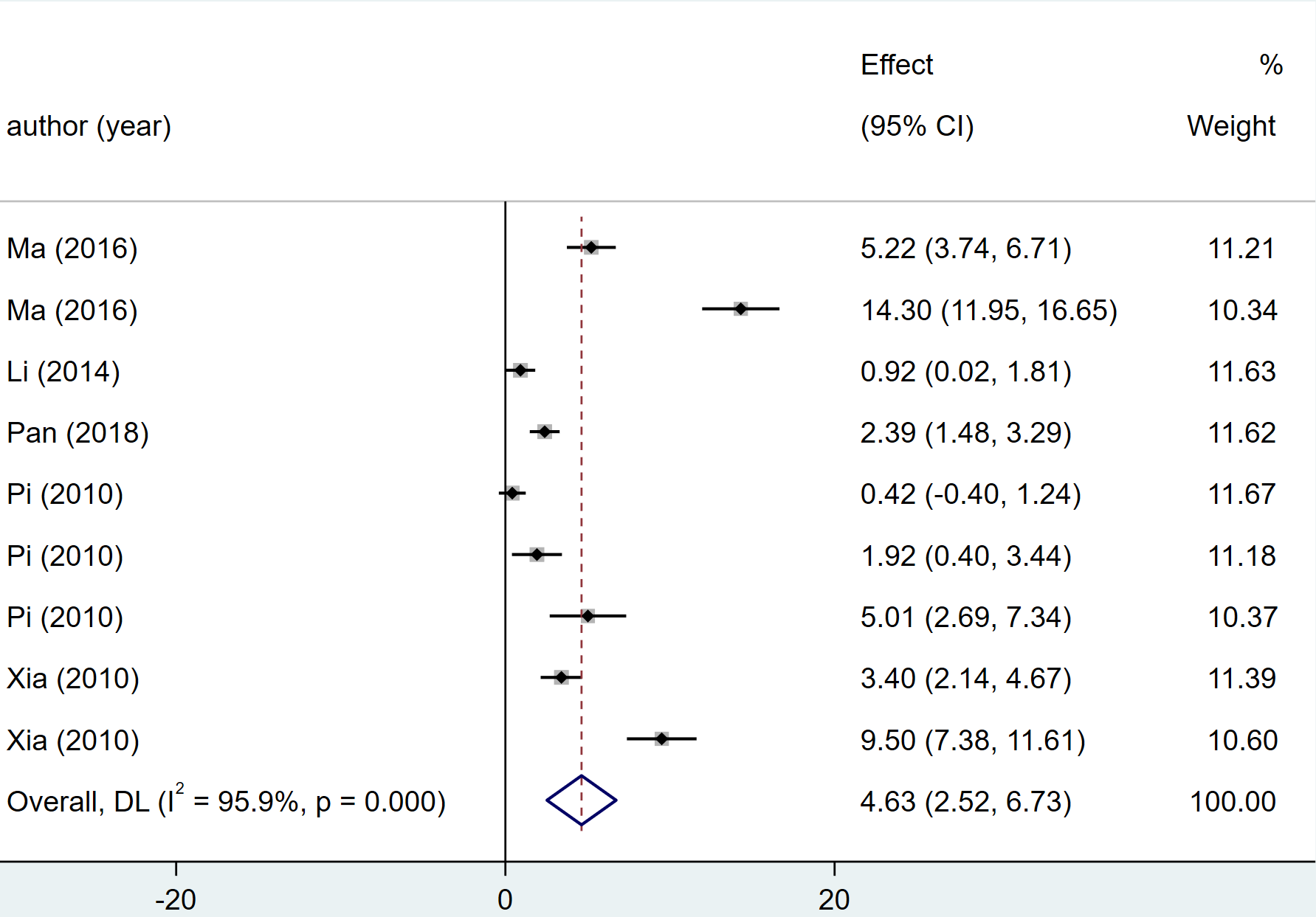
**

**Table 2.** Prevalence of myopia and proportion of low myopia, moderate myopia and high myopia in rural and urban settings

|  | **Rural** | | | **Urban** | | |
| --- | --- | --- | --- | --- | --- | --- |
|  | Value | Range for one-way sensitivity analysis | Distributions used in the probabilistic sensitivity analysis | Value | Range for one-way sensitivity analysis | Distributions used in the probabilistic sensitivity analysis |
| Prevalence of myopia | 5% | ±10%  (4.50%-5.50%) | Beta (379.95,7219.05) | 13% | ±10%  (11.70%-14.30%) | Beta (347.87,2328.05) |
| % of low myopia among myopia patients | 96.64%^18-20^ | ±10%  (86.98%-100.00%) | Beta (12.47,0.43) | a | a | a |
| % of moderate myopia among myopia patients | 1.47%^18-20^ | ±10%  (1.32%-1.62%) | Beta (394.11,26415.78) | a | a | a |
| % of high myopia among myopia patients | 1.89%^18-20^ | ±10%  (1.70%-2.08%) | Beta (392.42,20370.6) | a | a | a |

**Table 3.** Coverage, compliance, sensitivity and specificity of different intervention methods

| **Parameters** | **Subgroups** | **Rural** | | | **Urban** | | |
| --- | --- | --- | --- | --- | --- | --- | --- |
|  |  | Value | Range for one-way sensitivity analysis | Distributions used in the probabilistic sensitivity analysis | Value | Range for one-way sensitivity analysis | Distributions used in the probabilistic sensitivity analysis |
| Sensitivity of school-based myopia screening | By age (year) |  |  |  |  |  |  |
|  | 6 | 86.5%^21^ | Not defined | Not defined | a | a | a |
|  | 7-12 | 95.3%^21^ | Not defined | Not defined | a | a | a |
|  | >=13 | 97.2%^21^ | Not defined | Not defined | a | a | a |
| Specificity of school-based myopia screening | By age (year) |  |  |  |  |  |  |
|  | 6 | 93.8%^21^ | Not defined | Not defined | a | a | a |
|  | 7-12 | 92.5%^21^ | Not defined | Not defined | a | a | a |
|  | >=13 | 87.5%^21^ | Not defined | Not defined | a | a | a |
| Coverage of school-based vision test | NA | 91.8% (National Health Commission of the People’s Republic of China) | ±10%  (82.6%-100%) | Beta (31.88,2.85) | a | a | a |
| Compliance with hospital diagnosis examination | NA | 35.3%^22, 23^ | ±10%  (31.77%-38.83%) | Beta (258.45,473.7) | 73.3%^24^ | ±10%  (65.97%-80.63%) | Beta (106.07,38.64) |
| Coverage of | By interventions |  |  |  |  |  |  |
| Spectacles | School-based myopia screening strategy | 18.33%^25, 26^ | ±10%  (16.50%-20.60%) | Beta (326.5,1454.72) | 46.5%^25^ | ±10%  (41.85%-51.15%) | Beta (213.54,245.68) |
|  | Myopia prevention and control strategy | 36%^26, 27^ | ±10%  (32.40%-39.60%) | Beta (255.64,454.47) | 68.3%^28, 29^ | ±10%  (61.47%-75.13%) | Beta (126.12,58.53) |
| More outdoor activities | School-based myopia screening strategy | 22.73%^30^ | ±10%  (20.46%-25.00%) | Beta (308.85,1049.94) | a | a | a |
|  | Traditional myopia prevention and control strategy | 49.49%^30^ | ±10%  (44.54%-54.44%) | Beta (201.55,205.7) | a | a | a |
|  | Digital comprehensive myopia prevention and control strategy | 83.8%^31^ | ±10%  (75.42%-92.18%) | Beta (63.96,12.36) | a | a | a |

**Table 4.** Transition rate before and after interventions

| **Parameters** | **Subgroups** | **Value** | **Range for one-way sensitivity analysis** | **Distributions used in the probabilistic sensitivity analysis** |
| --- | --- | --- | --- | --- |
| Transition rate before intervention | By age |  |  |  |
| Non-myopia to low myopia^a^ | 6 | 8.8%^32^ | Not defined | Not defined |
|  | 7 | 16.4%^32^ | Not defined | Not defined |
|  | 8 | 16.1%^32^ | Not defined | Not defined |
|  | 9 | 15.5%^32^ | Not defined | Not defined |
|  | 10 | 16.5%^32^ | Not defined | Not defined |
|  | 11 | 18.2%^32^ | Not defined | Not defined |
|  | 12 | 20.6%^32^ | Not defined | Not defined |
|  | 13 | 36.3%^32^ | Not defined | Not defined |
|  | 14 | 16.3%^32^ | Not defined | Not defined |
|  | 15 | 11.3%^32^ | Not defined | Not defined |
|  | 16 | 16.7%^32^ | Not defined | Not defined |
|  | 17 | 15.4%^32^ | Not defined | Not defined |
|  | 18 | 13.8%^32^ | Not defined | Not defined |
| Low to moderate myopia | 6-10 | 22.82%^33^ | Not defined | Not defined |
|  | 11-15 | 20.33%^33^ | Not defined | Not defined |
|  | 16-18 | 9.65%^33^ | Not defined | Not defined |
| Low to high myopia | 6-10 | 9.73%^33^ | Not defined | Not defined |
|  | 11-15 | 8.73%^33^ | Not defined | Not defined |
|  | 16-18 | 4.27%^33^ | Not defined | Not defined |
| Moderate to high myopia | 6-10 | 18.63%^33^ | Not defined | Not defined |
|  | 11-15 | 16.64%^33^ | Not defined | Not defined |
|  | 16-18 | 7.98%^33^ | Not defined | Not defined |
| Incidence risk with health education | NA | 80%^31, 34, 35^ | ±10%  (72%-88%) | Beta (79.2,19.8) |
| Reduction in myopia progression with intervention | NA | 67%^36^ | ±10%  (60.30%-73.70%) | Beta (131.33,64.68) |

^a^ In studies where multi-year rather than 1-year incidence was reported, the 1-year incidence was calculated using the formula r=−ln(1−p)/t, where r denotes the 1-year incidence and p represents the cumulative incidence over length of interval t (Rothman KJ. Epidemiology: an introduction. 2nd edn. New York, NY: Oxford University Press, 2012)

**Table 5.** Utility, disability weight and other parameters

| **Parameters** | **Subgroups** | **Value** | **Range for one-way sensitivity analysis** | **Distributions used in the probabilistic sensitivity analysis** |
| --- | --- | --- | --- | --- |
| Utilities | By stage |  |  |  |
|  | Normal | 1^37^ | 90.00%-100.00% | Beta (-1,0) |
|  | Low myopia | 0.96^38^ | 95.00%-97.00% | Beta (15.04,0.63) |
|  | Moderate myopia | 0.96^38^ | 95.00%-97.00% | Beta (15.04,0.63) |
|  | High myopia | 0.95^38^ | 94.00%-96.00% | Beta (19.05,1) |
| Disability weight | By stage |  |  |  |
|  | Normal | 0^39^ | ±10%  (0.27%-0.33%) | Beta (398.8,132533.54) |
|  | Low myopia | 0.003^39^ | ±10%  (0.27%-0.33%) | Beta (398.8,132533.54) |
|  | Moderate myopia | 0.003^39^ | ±10%  (2.79%-3.41%) | Beta (387.57,12114.66) |
|  | High myopia | 0.031^39^ |  |  |
| Discount rate for costs and benefits | NA | 3.5%^40^ | Not defined | Not defined |
| Mortality rates | By age |  |  |  |
|  | 6-9 | 0.044%^41^ | Not defined | Not defined |
|  | 10-14 | 0.031%^41^ | Not defined | Not defined |
|  | 15-18 | 0.034%^41^ | Not defined | Not defined |

**Table 6**. Cost computation of school-based vision test

|  | **Annualized cost ($/year) in rural setting** | **Annualized cost ($/year) in urban setting** |
| --- | --- | --- |
| Training fee and allowance for teachers and school doctors^a^ | 339 | 423 |
| Equipment for school vision test^b^ | 2150 | 2150 |
| Wages for teachers and school doctors^c^ | 1415 | 1898 |
| Total number of schoolchildren in every screening | 1835 | 1835 |
| Annual cost per student (two tests a year) | 1.06 | 1.22 |

^a^ Teachers and school doctors are trained in local medical institutions, each training lasts for half a day. ^b^ The annualized cost for fixed assets was calculated by assuming a life span of 5 years and no salvage value. Screening projects including visual acuity and autorefractor, the equipment costs in 2021 is adjusted to the 2014 data for an annual interest rate of 5%. ^c^ The screening time is 1.5 minutes per student, and the total time per screening is 46 hours.

**Table 7**. Cost computation of traditional health education

|  | **Annualized cost ($/year) in rural setting** | **Annualized cost ($/year) in urban setting** |
| --- | --- | --- |
| Training fee and allowance for teachers^a^ | 216 | 258 |
| Publicity fee^b^ | 605 | 605 |
| Total number of children in every screening | 1835 | 1835 |
| Annual cost per student | 0.45 | 0.47 |

^a^ Teachers receive health education related knowledge training twice a year. ^b^ Publicity avenues include posters, videos, brochures and themed events to students and parents.

**Table 8**. Cost computation of digital health education

|  | **Annualized cost ($/year) in rural setting** | **Annualized cost ($/year) in urban setting** |
| --- | --- | --- |
| Training fee and allowance for teachers^a^ | 216 | 258 |
| Publicity fee ($/per person)^b^ | 11.74 | 11.74 |
| Total number of children in every screening | 1835 | 1835 |
| Annual cost per student | 11.86 | 11.88 |

^a^ Teachers receive health education related knowledge training twice a year. ^b^ Publicity avenues include sending text messages about myopia via Wechat and SMS to parents and students regularly.

**Table 9.** Cost computation of medical intervention

|  | **Annualized cost ($/year) in rural setting** | **Annualized cost ($/year) in urban setting** |
| --- | --- | --- |
| Direct medical cost^a^ | 149 | 149 |
| Direct non-medical cost^b^ | 6 | 6 |
| Indirect cost^c^ | 82 | 110 |
| Annual cost per student | 237 | 265 |

^a^ Students visit medical institutions twice a year. According to the *Report on China's Eyeglasses Industry*, students replace an average pair of glasses every year. ^b^ Direct non-medical costs include transportation and meals for one student and one parent. ^c^ Indirect costs include lost wages for one parent. The average daily income for rural and urban residents is $41 and $55, respectively.

**Figure 5.** Deterministic 1-way sensitivity analysis


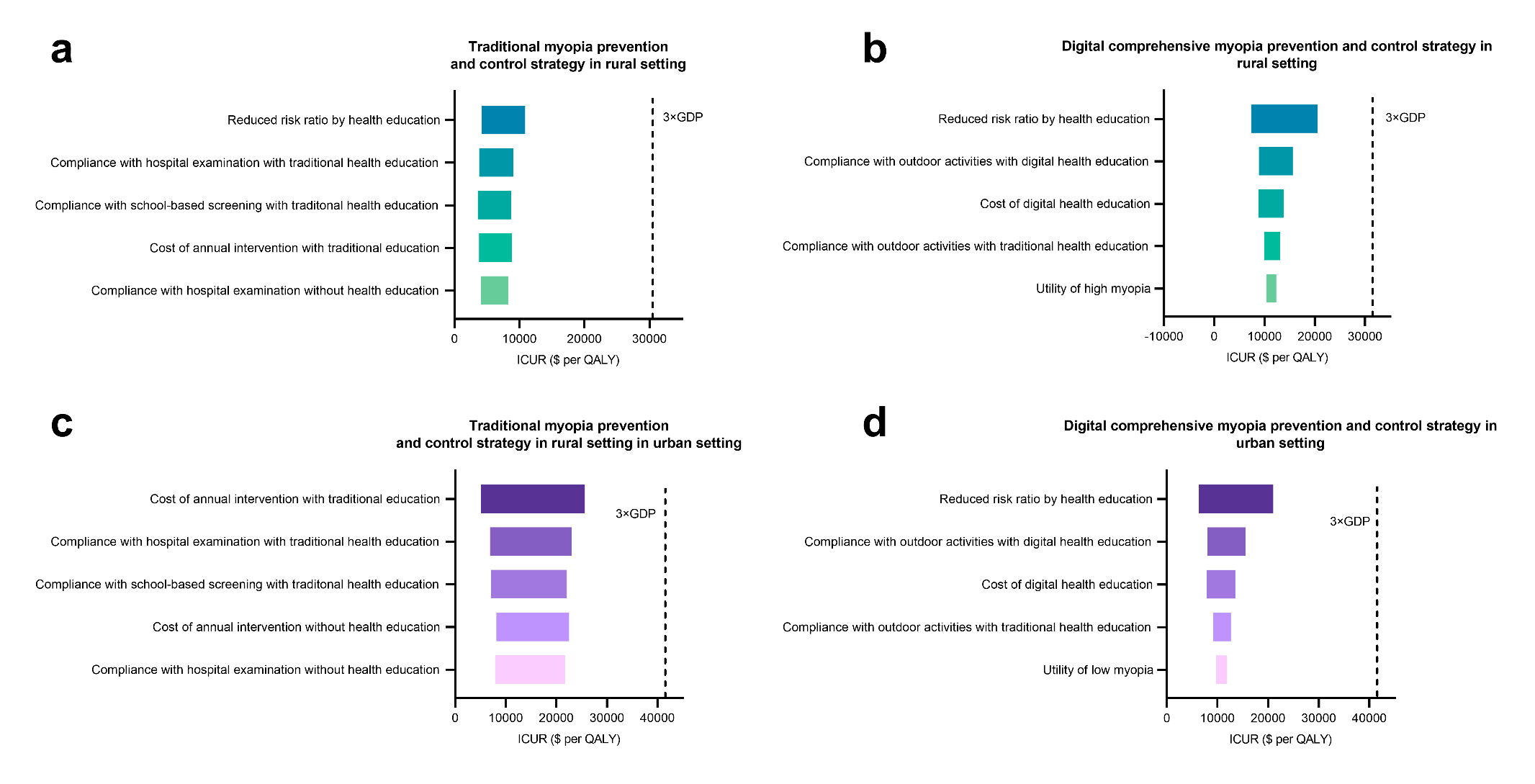


Costs are given in US dollars. QALY=quality-adjusted life-year. ICUR=incremental cost-utility ratio. The top 5 parameters which caused the greatest impact on ICURs are shown. The cost-effectiveness threshold was $30501 in rural setting and $41568 in urban setting.

**Figure 6.** Results of probabilistic sensitivity analysis

**
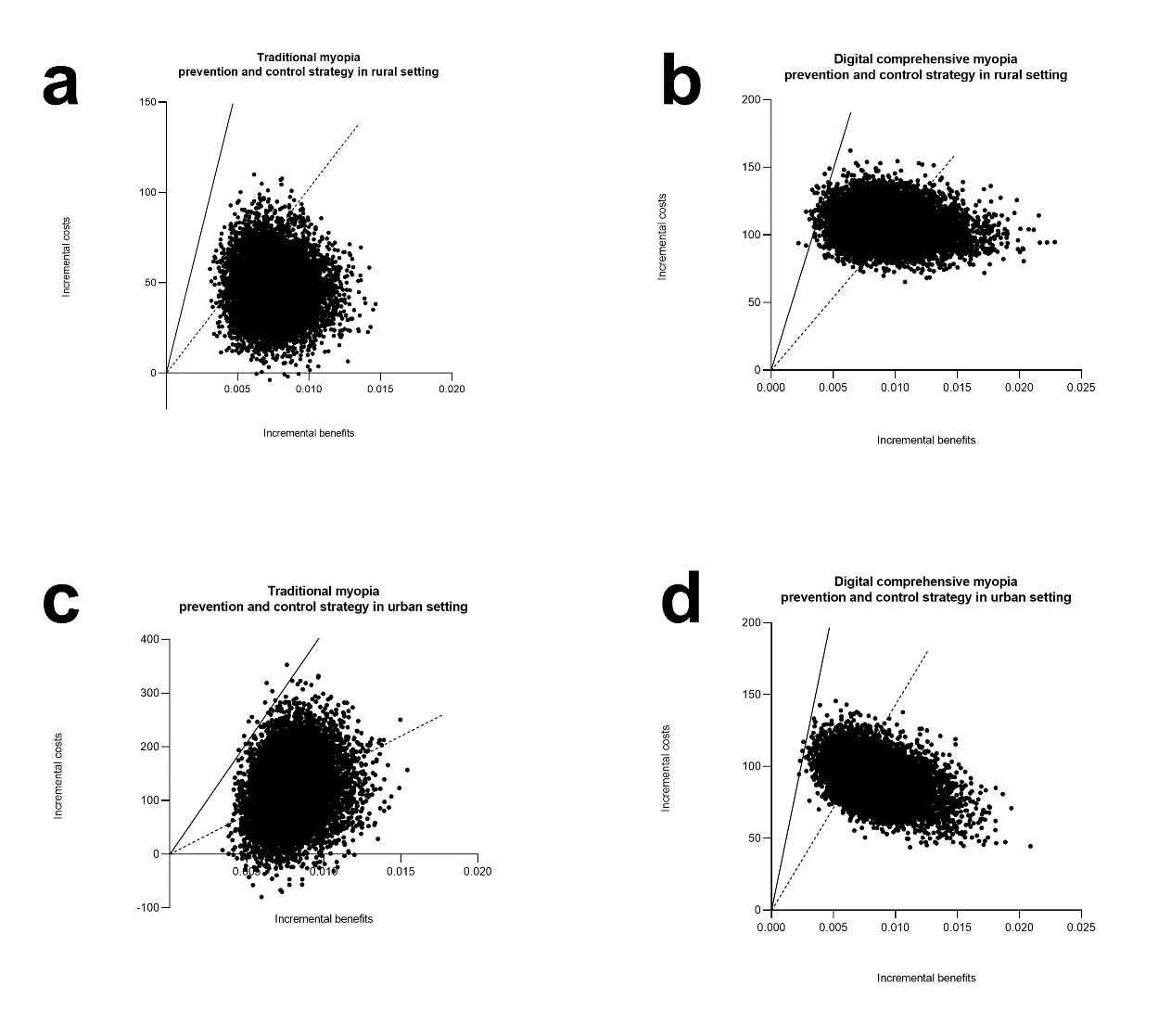
**

Probabilistic sensitivity analysis was conducted by taking 10,000 random draws. Dashed and solid lines represent one-time and three-times GDP.

**Figure 7.** Acceptability curve of school-based myopia screening strategy, traditional myopia prevention and control strategy and digital comprehensive myopia prevention and control strategy in rural and urban settings

**
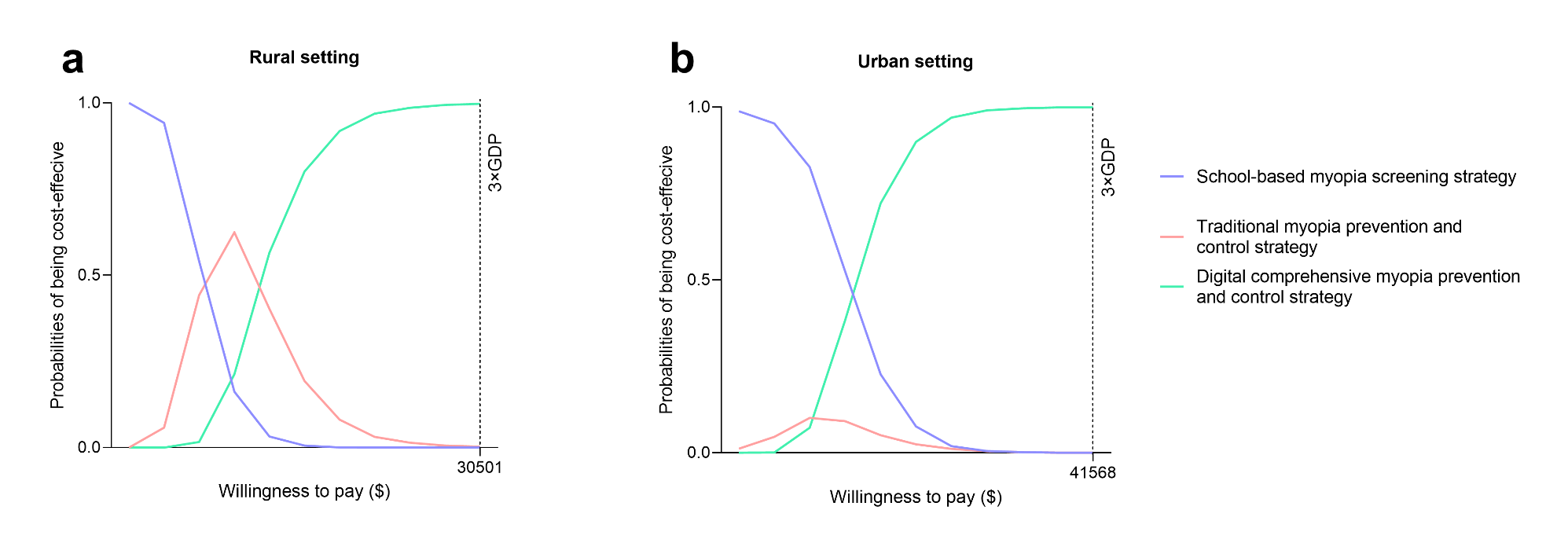
**

**Reference**

1 He M-g, Lin Z, Huang J, Lu Y, Wu C-f, Xu J-j. [Population-based survey of refractive error in school-aged children in Liwan District, Guangzhou]. *Zhonghua Yan Ke Za Zhi* 2008;**44**:491-6.

2 Liu L, Li H, Tang Z, Huang Z, Yang X. Prevalence of Refractive Error in School Children in Suining City of Sichuan Province, China: A School-Based Cross-Sectional Study. *Appl Bionics Biomech* 2022;**2022**:4845713.

3 Zhang XJ, Zhang Y, Kam KW, et al. Prevalence of Myopia in Children Before, During, and After COVID-19 Restrictions in Hong Kong. *JAMA Netw Open* 2023;**6**:e234080.

4 Zhang X, Cheung SSL, Chan H-N, et al. Myopia incidence and lifestyle changes among school children during the COVID-19 pandemic: a population-based prospective study. *Br J Ophthalmol* 2022;**106**:1772-8.

5 Yang Y, Li R, Ting D, et al. The associations of high academic performance with childhood ametropia prevalence and myopia development in China. *Ann Transl Med* 2021;**9**:745.

6 Li Y, Xing Y, Jia C, et al. Beijing Pinggu Childhood Eye Study: The Baseline Refractive Characteristics in 6- to 12-Year-Old Chinese Primary School Students. *Front Public Health* 2022;**10**:890261.

7 Hsieh M-W, Chang H-C, Chen Y-H, Chien K-H. Classification-Based Approaches to Myopia Control in a Taiwanese Cohort. *Front Med (Lausanne)* 2022;**9**:879210.

8 Leng L, Zhang J, Xie S, et al. Effect of Sunshine Duration on Myopia in Primary School Students from Northern and Southern China. *Int J Gen Med* 2021;**14**:4913-22.

9 Mu J, Zeng D, Fan J, et al. Epidemiological Characteristics and Influencing Factors of Myopia Among Primary School Students in Southern China: A Longitudinal Study. *Int J Public Health* 2023;**68**:1605424.

10 Lin T, Hu J, Lin J, Chen J, Wen Q. Epidemiological investigation of the status of myopia in children and adolescents in Fujian Province in 2020. *Jpn J Ophthalmol* 2023;**67**:335-45.

11 Yue Y, Liu X, Yi S, Liu B, Yi H, Li H. High prevalence of myopia and low hyperopia reserve in 4411 Chinese primary school students and associated risk factors. *BMC Ophthalmol* 2022;**22**:212.

12 Ye L, Yang Y-Q, Zhang G-Y, et al. Increasing prevalence of myopia and the impact of education in primary-school students in Xi'an, north-western of China. *Front Public Health* 2022;**10**:1070984.

13 Ma Y, Qu X, Zhu X, et al. Age-Specific Prevalence of Visual Impairment and Refractive Error in Children Aged 3-10 Years in Shanghai, China. *Invest Ophthalmol Vis Sci* 2016;**57**:6188-96.

14 Li Z, Xu K, Wu S, et al. Population-based survey of refractive error among school-aged children in rural northern China: the Heilongjiang eye study. *Clin Exp Ophthalmol* 2014;**42**:379-84.

15 Pan C-W, Wu R-K, Li J, Zhong H. Low prevalence of myopia among school children in rural China. *BMC Ophthalmol* 2018;**18**:140.

16 Pi L-H, Chen L, Liu Q, et al. Refractive status and prevalence of refractive errors in suburban school-age children. *Int J Med Sci* 2010;**7**:342-53.

17 Xia W, Gong L, Zhang X, et al. [Investigation analysis of children myopia in Qingpu district of Shanghai]. *International Eye Science* 2010;**10**:2157-8.

18 Li S-M, Li S-Y, Liu L-R, et al. Peripheral refraction in 7- and 14-year-old children in central China: the Anyang Childhood Eye Study. *Br J Ophthalmol* 2015;**99**:674-9.

19 Li S-M, Liu L-R, Li S-Y, et al. Design, methodology and baseline data of a school-based cohort study in Central China: the Anyang Childhood Eye Study. *Ophthalmic Epidemiol* 2013;**20**:348-59.

20 Qi Z, Chen J, He X, et al. [Epidemiology of high myopia among children and adolescents in China]. *Zhonghua Yan Ke Za Zhi* 2023;**59**:138-45.

21 Wang J, Xie H, Morgan I, et al. How to Conduct School Myopia Screening: Comparison Among Myopia Screening Tests and Determination of Associated Cutoffs. *Asia Pac J Ophthalmol (Phila)* 2022;**11**:12-8.

22 Wang H, Cousineau C, Fan Y, et al. Gender equity in vision care seeking behavior among caregivers: evidence from a randomized controlled trial in rural China. *Int J Equity Health* 2022;**21**:26.

23 Lin Z, Gao TY, Vasudevan B, et al. Near work, outdoor activity, and myopia in children in rural China: the Handan offspring myopia study. *BMC Ophthalmol* 2017;**17**:203.

24 Ma Y, Wen Y, Zhong H, et al. Healthcare utilization and economic burden of myopia in urban China: A nationwide cost-of-illness study. *J Glob Health* 2022;**12**:11003.

25 Zhou W, Feng X, Chen H, et al. [Prevalence of screening myopia and refractive correction among primary and middle school students in Xuzhou city]. *International Eye Science* 2022;**22**:647-51.

26 Ma X, Zhou Z, Yi H, et al. Effect of providing free glasses on children's educational outcomes in China: cluster randomized controlled trial. *BMJ* 2014;**349**:g5740.

27 Zhang Y, Guan H, Du K, et al. Effects of Vision Health Education and Free Eyeglasses on Knowledge of Vision and Usage of Spectacles Among Primary School Students: Evidence from Gansu and Shaanxi Provinces in China. *Risk Manag Healthc Policy* 2021;**14**:1449-64.

28 Yi H, Zhang H, Ma X, et al. Impact of Free Glasses and a Teacher Incentive on Children's Use of Eyeglasses: A Cluster-Randomized Controlled Trial. *Am J Ophthalmol* 2015;**160**.

29 Zeng Y, Keay L, He M, et al. A randomized, clinical trial evaluating ready-made and custom spectacles delivered via a school-based screening program in China. *Ophthalmology* 2009;**116**:1839-45.

30 Wu P-C, Chen C-T, Lin K-K, et al. Myopia Prevention and Outdoor Light Intensity in a School-Based Cluster Randomized Trial. *Ophthalmology* 2018;**125**:1239-50.

31 Li Q, Guo L, Zhang J, et al. Effect of School-Based Family Health Education via Social Media on Children's Myopia and Parents' Awareness: A Randomized Clinical Trial. *JAMA Ophthalmol* 2021;**139**:1165-72.

32 Chen J, He X, Wang J, et al. [Forcasting the prevalence of myopia among students aged 6-18 years in China from 2021 to 2030]. *Zhonghua Yan Ke Za Zhi* 2021;**57**:261-7.

33 Verkicharla PK, Kammari P, Das AV. Myopia progression varies with age and severity of myopia. *PLoS One* 2020;**15**:e0241759.

34 He M, Xiang F, Zeng Y, et al. Effect of Time Spent Outdoors at School on the Development of Myopia Among Children in China: A Randomized Clinical Trial. *JAMA* 2015;**314**:1142-8.

35 Li S-M, Ran A-R, Kang M-T, et al. Effect of Text Messaging Parents of School-Aged Children on Outdoor Time to Control Myopia: A Randomized Clinical Trial. *JAMA Pediatr* 2022.

36 Bao J, Yang A, Huang Y, et al. One-year myopia control efficacy of spectacle lenses with aspherical lenslets. *Br J Ophthalmol* 2022;**106**:1171-6.

37 Sankaridurg P, Tahhan N, Kandel H, et al. IMI Impact of Myopia. *Invest Ophthalmol Vis Sci* 2021;**62**:2.

38 Li S, Wang G, Xu Y, Gray A, Chen G. Utility values among myopic patients in mainland China. *Optom Vis Sci* 2014;**91**:723-9.

39 Hong CY, Boyd M, Wilson G, Hong SC. Photorefraction Screening Plus Atropine Treatment for Myopia is Cost-Effective: A Proof-of-Concept Markov Analysis. *Clin Ophthalmol* 2022;**16**:1941-52.

40 Tang J, Liang Y, O'Neill C, Kee F, Jiang J, Congdon N. Cost-effectiveness and cost-utility of population-based glaucoma screening in China: a decision-analytic Markov model. *Lancet Glob Health* 2019;**7**:e968-e78.

41 Zhang W, Wei M. [The Evaluation of the Mortality and Life Expectancy of Chinese Population]. *Ren Kou Xue Kan* 2016;**38**:18-28.
